# UV light-induced spatial loss of sialic acid capping using a photoactivatable sialyltransferase inhibitor†

**DOI:** 10.1039/d3cb00006k

**Published:** 2023-05-25

**Authors:** Sam J. Moons, Daniël L.A.H. Hornikx, Mikkel K. M. Aasted, Johan F.A. Pijnenborg, Matteo Calzari, Paul B. White, Yoshiki Narimatsu, Henrik Clausen, Hans H. Wandall, Thomas J. Boltje, Christian Büll

**Affiliations:** a Cluster for Molecular Chemistry, Institute for Molecules and Materials, Radboud University Nijmegen Nijmegen The Netherlands thomas.boltje@ru.nl; b Department of Biomolecular Chemistry, Institute for Molecules and Materials, Radboud University Nijmegen Nijmegen The Netherlands christian.bull@ru.nl; c Copenhagen Center for Glycomics, Department of Cellular and Molecular Medicine, Faculty of Health Sciences, University of Copenhagen Copenhagen Denmark

## Abstract

Sialic acids cap glycans displayed on mammalian glycoproteins and glycolipids and mediate many glycan-receptor interactions. Sialoglycans play a role in diseases such as cancer and infections where they facilitate immune evasion and metastasis or serve as cellular receptors for viruses, respectively. Strategies that specifically interfere with cellular sialoglycan biosynthesis, such as sialic acid mimetics that act as metabolic sialyltransferase inhibitors, enable research into the diverse biological functions of sialoglycans. Sialylation inhibitors are also emerging as potential therapeutics for cancer, infection, and other diseases. However, sialoglycans serve many important biological functions and systemic inhibition of sialoglycan biosynthesis can have adverse effects. To enable local and inducible inhibition of sialylation, we have synthesized and characterized a caged sialyltransferase inhibitor that can be selectively activated with UV-light. A photolabile protecting group was conjugated to a known sialyltransferase inhibitor (P-SiaFNEtoc). This yielded a photoactivatable inhibitor, UV-SiaFNEtoc, that remained inactive in human cell cultures and was readily activated through radiation with 365 nm UV light. Direct and short radiation of a human embryonic kidney (HEK293) cell monolayer was well-tolerated and resulted in photoactivation of the inhibitor and subsequent spatial restricted synthesis of asialoglycans. The developed photocaged sialic acid mimetic holds the potential to locally hinder the synthesis of sialoglycans through focused treatment with UV light and may be applied to bypass the adverse effects related to systemic loss of sialylation.

## Abstract 2

We report the synthesis of a photocaged sialyltransferase inhibitor that enables inducible and spatial expression of asialoglycans in human cells.

## Graphical abstract

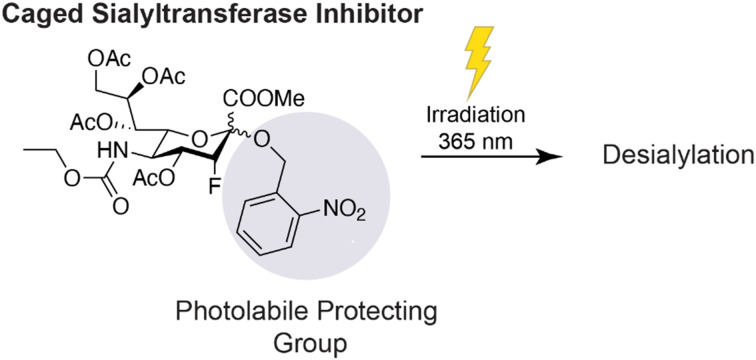

Sialic acids are a family of negatively charged nine-carbon sugars that are widely found in nature at the terminal position of glycans.^1,2^ In human cells, N-acetylneuraminic acid (Neu5Ac, SiaNAc) is the most abundant family member.^3^ Sialoglycans mediate numerous molecular interactions at the cell surface and serve as ligands for endogenous lectins such as the Siglec immune receptor family and exogenous lectins for example influenza haemagglutinin.^4–6^ Sialic acids play important roles in immune recognition events and immune modulation and form the cellular receptors for docking and entry for several human pathogens.^7^ Cancer cells often display altered glycosylation and especially changes in sialoglycans are frequently observed.^8^ The aberrant expression of sialoglycans in cancer likely derives from a combination of altered sialic acid biosynthesis, altered expression and activity of the 20 sialyltransferase isoenzymes, and the sialidases (NEU1-4) that add or remove sialic acids from glycans, respectively.^9^ Aberrant sialoglycan expression fosters tumor immune evasion through interactions with immune inhibitory Siglecs, and metastasis by facilitating binding to selectins.^10–13^

Targeting aberrant sialylation in cancer using small molecule inhibitors is emerging as a promising therapeutic approach.^14,15^ Paulson and co-workers have previously developed a cell-permeable metabolic inhibitor of sialylation based on peracetylated 3-fluorosialic acid (P-SiaFNAc).^16^ Upon entering the cell, P-SiaFNAc is deacetylated by esterases and converted into the active nucleotide sugar CMP-SiaFNAc by the CMP-sialic acid synthetase (CMAS). CMP-SiaFNAc acts as a direct competitive inhibitor of the sialyltransferase isoenzymes and indirectly, accumulation of CMP-SiaFNAc triggers feedback inhibition of the *de novo* sialic acid biosynthesis pathway from N-acetylmannosamine (ManNAc). Together, this effectively depletes all sialic acid capping in cells.^16,17^ This sialic acid mimetic is well-tolerated by mammalian cells and thus highly useful to probe the involvement of sialoglycans in biological processes.^18–22^

We and others have shown that P-SiaFNAc inhibits all sialylation including α2-3/6/8 linkages to the different glycoconjugates in mammalian cells. Treatment of cancer cells resulted in reduced tumor growth and metastasis in mouse models.^23–26^ The use of P-SiaFNAc has also revealed how sialoglycans influence antigen-presenting cell interactions with CD8^+^ T cells which may enable potentiating anti-tumor immune responses.^27,28^ We have recently developed more potent analogues of P-SiaFNAc by taking advantage of the substrate tolerance of the sialoglycan biosynthesis enzymes for C-5 substitutions of sialic acid,.^29–31^ We found that C-5 carbamate derivatives of P-SiaFNAc, *e.g.* P-SiaFNEtoc have about 30-times enhanced inhibitory potency. The higher inhibitory potency was attributed to increased metabolic flux towards the active CMP derivative and higher intracellular concentrations of the active CMP-activated inhibitor resulting in potent and long-lasting (>5 days) inhibition of sialylation.^31^

The major challenge for the *in vivo* use of these inhibitors and their potential application as anti-cancer drugs is to selectively deliver them to target tissues and cell types.^32^ Systemic administration of high doses of P-SiaFNEtoc in mice resulted in desialylation of most tissues leading to altered lymphocyte trafficking and liver and kidney damage.^23,33^ We have previously shown selective targeting of P-SiaFNAc to metastatic tumor cells using PLGA-based nanoparticles, but this approach will likely not allow targeting to solid tumors that are largely impenetrable for nanoparticles.^24^ An alternative solution are sialidase-antibody conjugates targeted to surface cancer antigens that locally hydrolyze sialic acids.^34^ However, the sialidase moiety in this system is constitutively active which could also lead to adverse effects and surface desialylation with sialidase recovers within several hours. A comparison of tumor outgrowth between melanoma cells pretreated with P-SiaFNEtoc or sialidase before injection into mice showed that the metabolic inhibition reduced tumor outgrowth significantly stronger.^24,25^ This suggests that metabolic sialylation inhibitors could have beneficial therapeutic effects due to their long-lasting effect.

To enable selective and locally-controlled inhibition of sialic acid capping with these inhibitors, we have explored the possibility to cage the sialylation inhibitor P-SiaFNEtoc with a photolabile *ortho*-nitrobenzyl group that has previously been employed to enable light-controlled metabolic processing of monosaccharides.^35,36^ We report the synthesis and characterization of a photoactivatable fluorosialic acid derivative, UV-SiaFNEtoc (Fig. 1). We show controlled photoactivation using 365 nm UV light and similar inhibitory potency *in vitro* compared to the parental uncaged fluorosialic acid. Focused UV-light treatment resulted in activation of the inhibitor and local synthesis of asialoglycans in irradiated HEK293 cells growing in monolayers without affecting viability. Altogether, this photoactivatable sialic acid mimetic may provide a potent strategy for inducible and local inhibition of sialoglycan biosynthesis in target cells or tissues using UV light.

**Fig. 1:**
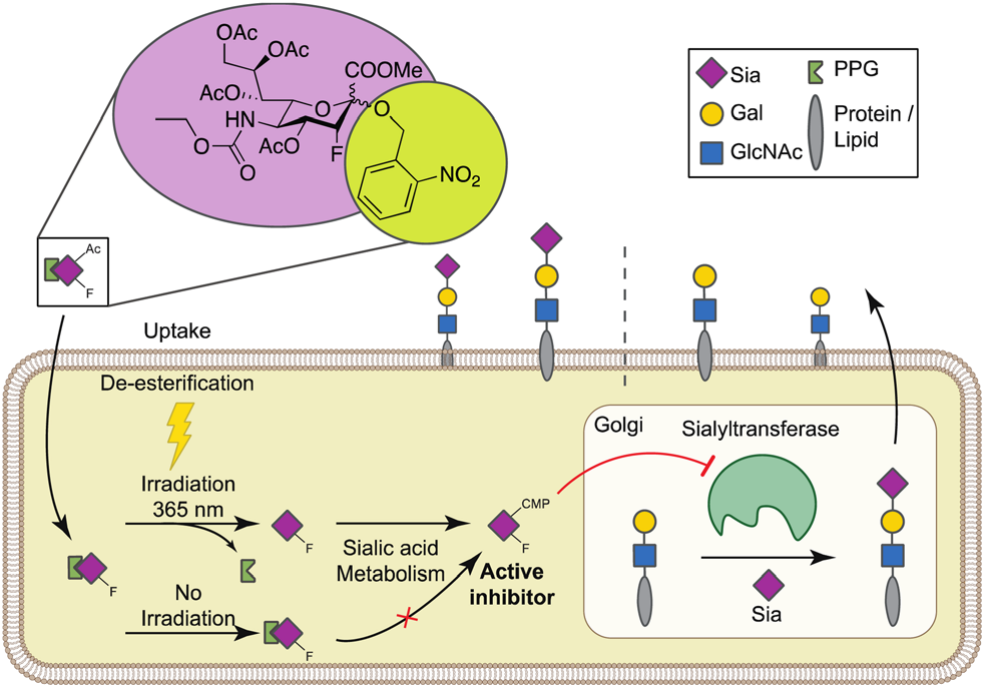
Graphical depiction of the UV-activatable sialylation inhibitor. After passive diffusion over the cell membrane and de-esterification, UV-SiaFNEtoc (4) can be activated with 365 nm UV light which cleaves the photolabile protecting group (PPG). Released SiaFNEtoc is converted into the active nucleotide sugar CMP-SiaFNEtoc that blocks Golgi-resident sialyltransferases and thus sialic acid capping. Without irradiation, the PPG remains intact and 4 is not recognized as substrate the sialic acid pathway. Sia = sialic acid, Gal = galactose, GlcNAc = N-acetyl glucosamine.

## Results and discussion

### Synthesis of a photocaged sialyltransferase inhibitor

**Scheme 1:**
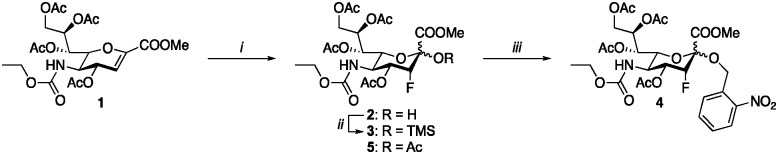
Synthesis of caged inhibitor 4. (i) Selectfluor, DMF, H_2_O, 64%; (ii) TMSNEt2, DCM, 80% (based on recovery); (iii) 2-nitrobenzyl bromide, CsF, DMF, 75%.

The synthesis of the peracetylated sialyltransferase inhibitor 5 (P-SiaFNEtoc) was described previously and this active inhibitor was used as a positive control (Scheme 1).^31^ To be able to selectively prevent sialic acid capping in mammalian cells using photoactivation, 5 was equipped with an *ortho*-nitrobenzyl ether at the C-2 position. This position is essential for conversion into the active CMP derivative of pro-drug 5 by CMAS. Attempts to directly turn glucal 1 into target inhibitor 4 were unsuccessful, so we opted to form the *ortho*-nitrobenzyl glycoside after fluorohydroxylation (2). To this end, compound 2 was prepared and reacted with *ortho*-nitrobenzyl bromide in a two-step procedure.^37^ The low reactivity of the anomeric alcohol required silylation using *N*,*N*-diethylaminotrimethylsilane and afforded trimethylsilyl compoud 3. Following a similar procedure described by Suzuki *et al.*, 3 was treated with CsF to selectively generate the cesium salt, which was then transformed into the nitrobenzyl glycoside by using 2-nitrobenzyl bromide yielding 4.^38^

### 365 nm light irradiation yields active sialylation inhibitor

**Fig. 2:**
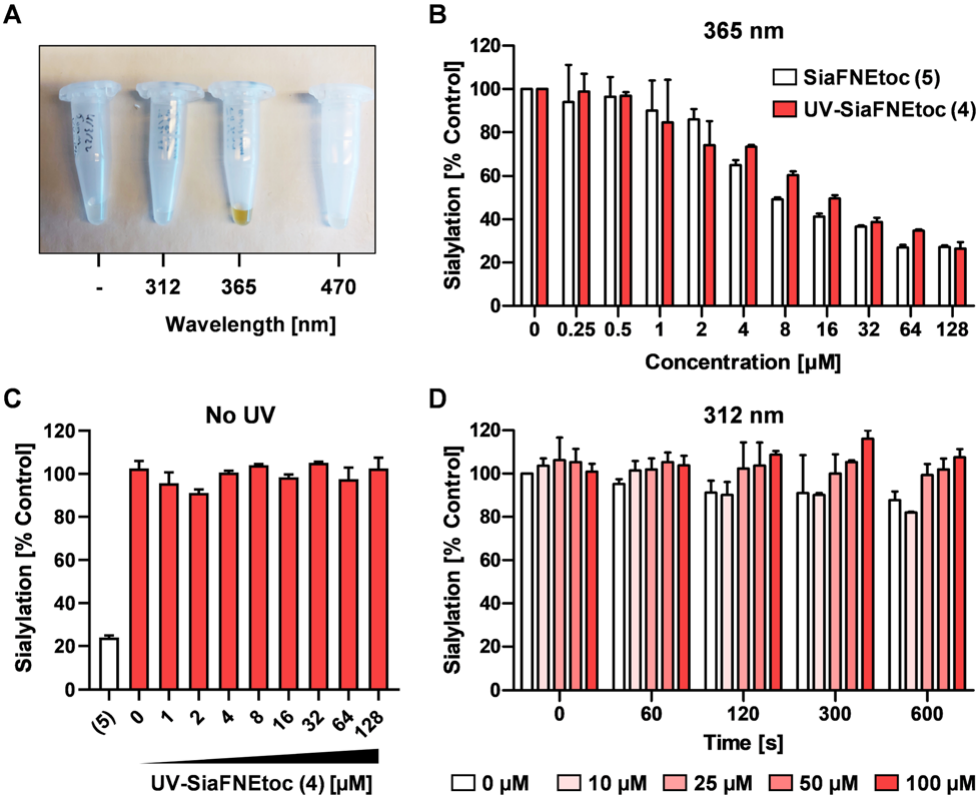
365 nm UV-light activation of 4. (A) Representative picture shows test tubes containing 4 irradiated with no, 312 nm, 365 nm, or 470 nm light for 900 sec. Brown coloration was observed only for 365 nm treatment. (B) Cell surface sialylation of HEK293 cells cultured for 3 days with increasing concentrations of 5 or 365 nm (300 sec) treated 4. (C) Sialylation of HEK293 cells treated for 3 days with increasing concentrations of non-irradiated 4 or 100 μM 5. (D) 5 was irradiated for 0–600 sec with a 312 nm light source and increasing concentrations were added to HEK293 cells for 3 days. Cell surface sialylation was quantified by flow cytometry using biotin**-**Pan-Lectenz-streptavidin Alexa Fluor 647 complexes. Bar diagrams show mean percentages Pan-lectenz binding (sialylation) ± SD normalized to untreated control cells of 2–3 representative experiments.

To investigate the photoactivation properties of 4, it was exposed to LED sources emitting 312 nm, 365 nm, or 470 nm light for 15 min. Discoloration from clear to brown upon treatment with 365 nm light was noticeable, but not with the other wavelengths (Fig. 2A). The brown color presumably derives from reactions with the reactive nitrosoaldehyde side product in DMSO.^39^ To assess its effect on sialylation, increasing concentrations of 4 irradiated 300 sec with 365 nm light were added to HEK293 cell cultures and equimolar concentrations of active inhibitor 5, not exposed to UV light, were added as positive control. Since SiaFNEtoc inhibits the *de novo* biosynthesis of sialoglycans, the inhibitory effect is best observed after 3 days when residual sialoglycans are depleted through sialoglycan turnover and degradation. After 3 days of incubation, cells were stained with Pan-Lectenz–streptavidin Alexa Fluor 647 complexes that bind sialoglycans and analyzed by flow cytometry. A concentration-dependent reduction of sialylation was observed in cells treated with 4 and 5 with 50% reduction of sialylation at 16 μM and maximum inhibition (80%) at 64 μM for both compounds (Fig. 2B). Higher concentrations did not further reduce Lectenz binding and residual binding was probably due to remaining non-sialic acid-mediated interactions.^16^ Notably, no effect on cell proliferation and viability was observed with concentrations of 4 up to 128 μM and similar inhibitory effects were observed in HeLa cell cultures (Fig. S1, ESI†). Compounds 4 and 5 displayed similar inhibitory potency suggesting that 4 was efficiently deprotected after the 365 nm treatment. Radiation of 5 showed no change in inhibitory potency, suggesting that the sialic acid mimetic is stable (Fig. S2, ESI†). To ensure that 4 requires activation by UV treatment, cells were incubated with 0–128 μM of non-irradiated 4 for 3 days and no reduction in sialylation was detectable (Fig. 2C). This shows that 4 remains inactive in cell cultures without exposure to 365 nm UV light. It has been demonstrated that anomeric 2-nitrobenzyl glycosides are labile upon irradiation at wavelengths longer than 320 nm.^35^

To demonstrate that a specific wavelength is required for uncaging, 4 was irradiated with 312 nm light for up to 600 sec and increasing concentrations were added to HEK293 cells for 3 days (Fig. 2D). No effect on cell surface sialylation was observed, suggesting that the specific wavelength of 365 nm is needed to cleave the photo-protecting group. This was also in line with our observation that only 365 nm treatment resulted in coloration of 4. LED-NMR analysis of 4 confirmed photolysis at 365 nm and formation of the active inhibitor (Fig. S3, ESI†). Notably, 4 showed an absorbance peak between 250–300 nm, but remained inactive when treated with 312 nm light (Fig. S4, ESI†). Presumably, the substituent may influence the absorption which has been reported for other nitrobenzyl derivatives while photolysis occurs most efficiently at 365 nm.^40^ Altogether, these data show that we have synthesized a photoactivatable sialylation inhibitor that can be specifically activated with 365 nm light.

### Intracellular UV light activation of UV-SiaFNEtoc

**Fig. 3:**
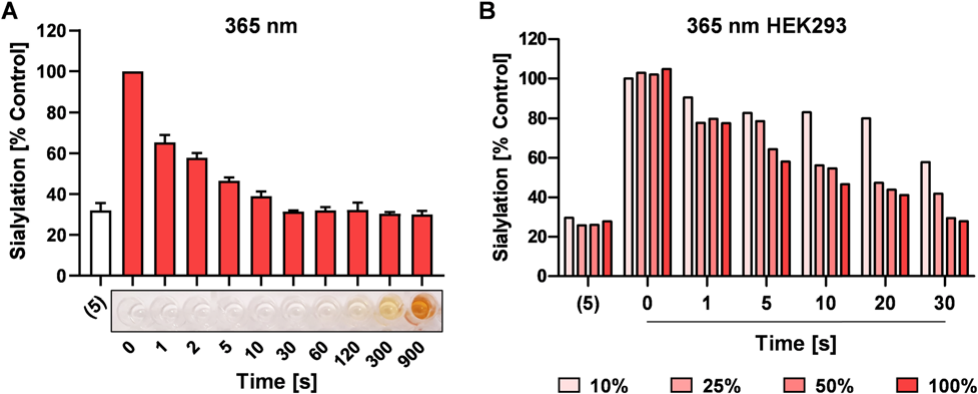
Activation of 4 in HEK293 cells. (A) 4 was irradiated with 365 nm light for 0–900 sec prior to addition of 100 μM to HEK293 cells followed by 3 days incubation and analysis of sialylation. 100 μM of 5 was added as positive control. Representative image shows coloration of 4 after 0–900 sec treatment with 365 nm light on a 96-well flat bottom plate. (B) 100 μM 4 was added to HEK293 cell cultures for 4 hours and after washing, the cells were directly irradiated for 0–30 sec with different intensities of 365 nm light (100% intensity corresponds to maximum intensity of the light source). After UV treatment, the cells were cultured for 3 days, and cell surface binding of biotin**-**Pan-Lectenz-streptavidin Alexa Fluor 647 complexes was measured by flow cytometry. Bar diagrams show mean percentages lectin binding (sialylation) ± SD normalized to untreated control cells of 2–3 representative experiments.

UV light exposure can result in DNA damage and depending on the dose and exposure time this damage is irreversible resulting in cytotoxicity.^41,42^ For potential application in cells, we next investigated the speed of photo-uncaging upon irradiation. **4** was irradiated with 365 nm light for different time points between 0 and 900 sec and 100 μM was added to HEK293 cells followed by analysis of sialylation using Pan-Lectenz or loss of sialic acid capping with PNA lectin after 3 days of culture (Fig. 3A and Fig. S5, ESI†). Already 1 sec pulse of 365 nm light resulted in *ca.* 25% reduced sialylation and a time-dependent inhibitory potency was observed. The maximum potency was reached after 30 sec of UV light treatment with resulted in similar reduction of sialylation compared with an equimolar concentration of **5**. This indicates that complete deprotection of **4** is achieved within 30 sec of radiation. Exposure of **4** for <60 sec to 365 nm light did not lead to coloration, but at longer exposure times brown color was formed (Fig. 3A). At 900 sec irradiation **4**, inhibited sialylation, but we also noticed reduced cell proliferation, presumably due to the formed nitrosoaldehyde.

Next, the possibility to activate 4 directly in cells was assessed. HEK293 cells growing in multi-well plates were pulsed for 4 hours with 100 μM of 4 or 5 and subsequently irradiated with different intensities of 365 nm light for 0–30 sec (Fig. 3B). After 3 days of culture, 20% decreased sialylation was observed already after 1 sec of max. exposure and 50% inhibition was detected after 10 sec of radiation with >25% intensity. Maximum inhibition (80%) was observed after 30 sec of irradiation. Importantly, exposure to 365 nm UV light for up to 30 sec was tolerated, but longer exposure times resulted in cell death 4 (Fig. S6, ESI†). UV energy measurements showed that 30 sec irradiation through tissue culture plastic at maximum intensity of the light source generated *ca.* 181 mW cm^−2^ (Table S1, ESI†). Half the intensity generated *ca.* 21.2 mW cm^−2^ and resulted in similar activation and inhibitory potency of 4. Together, this suggests that the UV treatment time and dose required for activation is compatible with live cells providing a treatment window for effective sialylation inhibition without impairing cell viability.

### Spatial inhibition of sialic acid capping in HEK293 monolayers

**Fig. 4:**
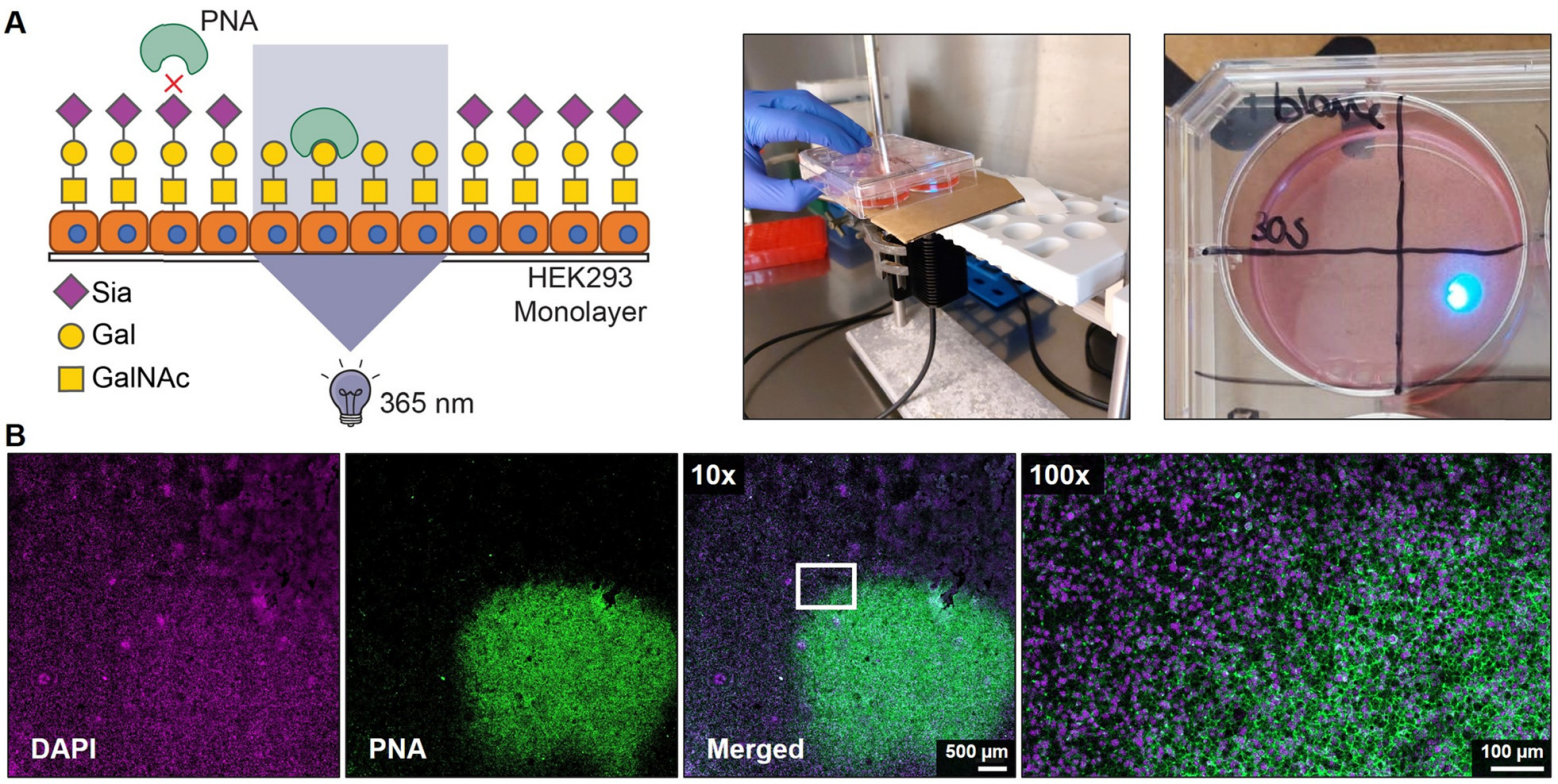
UV-induced asialoglycan synthesis in HEK293 cells. (A) Schematic representation (left) and pictures of experimental set-up (middle, right). HEK293 cells were grown as monolayer on cover slips in 6-well plates and a 365 nm UV source was installed below the sample with a paper filter to allow local irradiation of a *ca*. 5 × 5 mm area. HEK293 cells were pulsed with 150 μm with 4 for 4 hours followed by extensive washing to remove compound that has not been taken up. The coverslip was locally irradiated with 365 nm light for 30 seconds at maximum intensity and the cells were cultured for 48 h, fixed, and stained with PNA lectin that recognizes asialoglycans. (B) Representative confocal microscopy images show the irradiated area of the HEK293 monolayer pulsed with 4 and PNA (green) and DAPI (magenta) staining is shown at 10× magnification (left panels). The white rectangle indicates the irradiation border and a 100x magnification of that area is shown (right image).

Finally, we assessed the potential to locally activate **4** after uptake in cells. Monolayers of HEK293 cells were grown on a coverslip and pulsed for 4 hours with 100 μM of **4** followed by extensive washing to remove extracellular inhibitor. Next, a small area of *ca.* 5 × 5 mm of the coverslips was exposed for 30 sec to 365 nm light and 2 days later the monolayers were stained with PNA lectin that recognizes uncapped glycans terminating with galactose like the T antigen (Galβ1-3GalNAcα1-O-Ser/Thr) (Fig. 4A). No PNA binding was observed to unexposed areas which is in line with our flow cytometry data and literature showing that untreated HEK293 cells are not PNA reactive (Fig. S5, ESI†).^43^ Irradiated areas showed clear membrane staining with PNA suggesting that the local UV light treatment activated **4** inside the cell (Fig. 4B). DMSO-treated monolayers locally irradiated with 365 nm remained PNA-negative, thus UV light treatment alone did not result in PNA ligand formation (Fig. S7, ESI†). Importantly, no signs of cell death were observed in the irradiated areas and the nuclei remained regularly shaped (Fig. S8, ESI†). Noteworthy, exposure times for >30 sec inflicted damage to the cells and disruption of the monolayer. Altogether, these data show that the developed photocaged sialic acid mimetic **4** can be locally activated inside the cell with UV light enabling effective and spatial loss of sialic acid capping in cell culture systems.

## Conclusion

We have developed a photocaged derivative of the sialylation inhibitor P-SiaFNEtoc (5), UV-SiaFNEtoc (4) that can be activated upon treatment specifically with 365 nm light. Active inhibitor is obtained after short radiation with non-cytotoxic doses enabling light-inducible inhibition of cell surface sialylation. The developed approach was tolerated by human cell cultures and allowed effective inhibition of sialylation restricted locally to the UV light-treated area in a cell monolayer model. The photoactivatable sialylation inhibition approach may potentially be applied for example in superficial tumors penetrable by UV light, to modulate sialoglycans on cancer cells and evade immunosuppression safely.

## Author contributions

S.J.M, D.L.H., T.J.B. and C.B. conceived and designed the study. M.M.A., Y.N., H.C. and H.H.W. contributed to the cell radiation study. J.F.P. contributed to the chemical design and synthesis, and M.C. and P.B.W. contributed to the LED-NMR experiments. S.J.M, D.L.H., T.J.B. and C.B. wrote the manuscript. All authors approved the final version.

## Conflicts of interest

There are no conflicts to declare.

## Supplementary Material

CB-004-D3CB00006K-s001
