# Supplementary material for: UV light-induced spatial loss of sialic acid capping using a photoactivatable sialyltransferase inhibitor
Source: RSC Chem Biol. 2023 May 25;4(7):506–11. doi: 10.1039/d3cb00006k (PMC10320844; doi:10.1039/d3cb00006k)
Supplement: CB-004-D3CB00006K-s001 [file CB-004-D3CB00006K-s001.pdf]

Electronic Supplementary Material (ESI) for RSC Chemical Biology.
This journal is © The Royal Society of Chemistry 2023

SUPPORTING INFORMATION

UV light-induced spatial loss of sialic acid capping using a photoactivatable sialyltransferase inhibitor

Sam J. Moons,[a#] Daniël L.A.H. Hornikx,[b#] Mikkel M. Andersen,[c] Johan F.A. Pijnenborg,[a] Matteo Calzari,[a] Paul B. White,[a] Yoshiki Narimatsu,[c] Henrik Clausen,[c] Hans H. Wandall,[c] Thomas J. Boltje,*[a] Christian Büll,*[bc]

a. Cluster for Molecular Chemistry, Institute for Molecules and Materials, Radboud University Nijmegen, Nijmegen, The Netherlands.

b. Department of Biomolecular Chemistry, Institute for Molecules and Materials, Radboud University Nijmegen, Nijmegen, The Netherlands.

c. Copenhagen Center for Glycomics, Department of Cellular and Molecular Medicine, Faculty of Health Sciences, University of Copenhagen, Copenhagen, Denmark

[#] These authors contributed equally.

* To whom correspondence may be addressed: *thomas.boltje@ru.nl* and *christian.bull@ru.nl*

Electronic Supplementary Information (ESI) available: [details of any supplementary information available should be included here]. See DOI: 10.1039/x0xx00000x

Table of content

S2 – S3 General methods for synthesis

S4 Experimental methods

S5 – S13 Supplementary table and figures

S14 – S16 NMR spectra

General methods for synthesis

Chemicals were purchased at Sigma Aldrich, TCI Europe, Fisher Scientific, Fluorochem or Carbosynth and used without further purification. NMR spectra were recorded on a Bruker 400 MHz AVANCE IIIHD nanobay or a Bruker 500 MHz AVANCE III spectrometer equipped with a BBFO and Prodigy BB cryoprobe, respectively, and the compounds were assigned using ^{1}H NMR,^{13}C NMR, ^{19}F NMR, COSY, HSQC and HMBC spectra. Chemical shifts were reported in parts per million (ppm.) relative to reference ($CDCl_3$: ^{1}H: 7.26 ppm. and ^{13}C 77.16 ppm. NMR data are presented in the following way: chemical shift, multiplicity (s = singlet, bs = broad singlet, d = doublet, t = triplet, dd = doublet of doublets, m = multiplet and/or multiple resonances) and coupling constants J in Hz. ^{13}C spectra are taken as APT experiments, in which CH / CH_3 multiplicities are up and C / CH_2 / solvent multiplicities are down. Reactions were monitored using TLC F_{254} (Merck KGaA) using UV absorption detection (254 nm) and by spraying them with potassium permanganate or 10% conc. H_2SO_4 in MeOH or cerium ammonium molybdate stain (Hannesian's stain) followed by charring at 300°C. Mass spectra were recorded on a JEOL AccuTOF CS JMS-T100CS (ESI) mass spectrometer. Purification by flash column chromatography was executed using automatic flash column chromatography on a Biotage Isolera Spektra One using Silicycle cartridges (Biotage, 30-100 µm, 60 Å) 4-12 g. Reactions under protective atmosphere were performed under positive Ar/N_2 flow in flame-dried flasks. Reactions were performed at room temperature unless stated otherwise.

Methyl 3,5-dideoxy-5-[(ethoxycarbonyl)amino]-3-fluoro-4,7,8,9-tetra-*O*-acetyl-D-erithro-a,β-L-manno-non-2-ulopyranosonate(2)

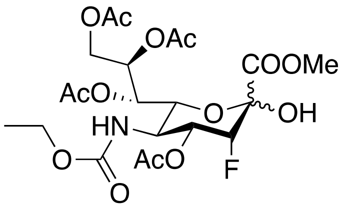

1 (1.104 g, 2.19 mmol, 1 eq.) was dissolved in DMF (16 mL, 91 eq.) and H_2O (5.2 mL, 131 eq.). Selectfluor (4 eq.) was added, and the reaction mixture was stirred at 50°C for three days. The mixture was concentrated, dissolved in EtOAc, washed with H_2O, brine, and dried using $MgSO_4$. The crude product was concentrated and purified using flash column chromatography (0 – 70% EtOAc in heptane) to afford the product (760 mg, 64%)

^{1}H NMR (500 MHz, $CDCl_3$) δ 5.50 – 5.29 (m, 3H), 5.02 – 4.87 (m, 2H), 4.78 (d, *J* = 12.3 Hz, 1H), 4.28 (d, *J* = 10.6 Hz, 1H), 4.20 – 4.03 (m, 4H), 3.88 (s, 3H), 2.18 (s, 3°CH), 2.11 – 2.11 (m, 6H), 2.06 (s, 3H), 1.23 (t, *J* = 7.1 Hz, 3H). **^{13}C NMR** (126 MHz, $CDCl_3$) δ 171.2, 170.5, 170.3, 167.6, 155.9, 94.3, 94.1, 87.7, 86.2, 71.5, 71.0, 69.6, 69.5, 68.3, 62.9, 61.4, 53.4, 47.0, 21.0, 20.8, 20.8, 20.7, 14.5. **HRMS** (ESI+) m/z calculated for $C_{21}H_{30}NO_{14}FNa$ 562.1548, found 562.1535. **TLC** (EtOAc/Heptane 50/50, v/v) R_f = 0.2

Methyl 3,5-dideoxy-5-[(ethoxycarbonyl)amino]-3-fluoro-4,7,8,9-tetra-*O*-acetyl-2-trimethylsilyl-D-erithro-a,β-L-manno-non-2-ulopyranosonate (3)

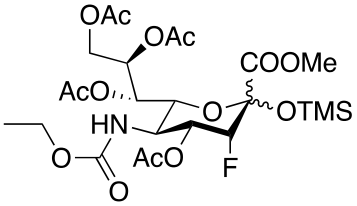

2 (310 mg, 0.58 mmol, 1 eq.) was dissolved in DCM (0.1M) and cooled to 0°C. $TMSNEt_2$ (2.19 mL, 20 eq.) was added. The mixture was stirred for 1h, warmed up to rt and stirred overnight. The mixture was concentrated and purified using flash column chromatography (0 – 50% EtOAc in heptane) to afford the product (160 mg, 46%) and recovered starting material (106mg, 34%).

^{1}H NMR (500 MHz, $CDCl_3$) δ 5.49 (dd, *J* = 3.5, 2.3 Hz, 1H), 5.45 – 5.33 (m, 1H), 5.24 (dt, *J* = 8.3, 2.7 Hz, 1H), 5.00 – 4.86 (m, 1H), 4.82 (d, *J* = 12.3 Hz, 1H), 4.51 (d, *J* = 9.8 Hz, 1H), 4.28 – 4.14 (m, 2H), 4.14 – 4.01 (m, 3H), 3.85 (s, 3H), 2.18 (s, 3H), 2.12 (s, 3H), 2.10 (s, 3H), 2.06 (s, 3H), 1.23 (t, *J* = 7.1 Hz, 3H), 0.22 (s, 9H). ^{13}C NMR (126 MHz, $CDCl_3$) δ 170.7, 170.6 170.5, 170.3, 167.0, 155.7, 95.5, 95.3, 88.8, 87.3, 77.3, 77.0, 76.8, 72.5, 71.9, 62.9, 62.8,

61.4, 53.0, 46.8, 29.7, 21.0, 20.8, 20.8, 20.7, 14.5, 0.6. **HRMS** (ESI+) m/z calculated for $C_{24}H_{38}NO_{14}FSiNa$ 634.1943, found 634.1921. **TLC** (EtOAc/Heptane 50/50, v/v) R_f = 0.4

Methyl 3,5-dideoxy-5-[(ethoxycarbonyl)amino]-3-fluoro-2-(2-nitrobenzyl)-4,7,8,9-tetra-*O*-acetyl-D-erithro-

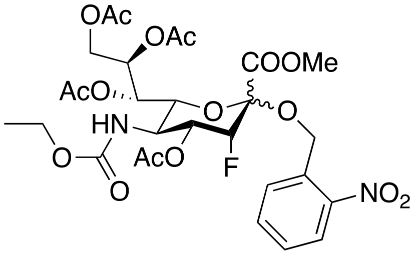

a,β-L-manno-non-2-ulopyranosonate (4)

3 (38 mg, 62 µmol, 1 eq.) was dissolved in dry DMF (0.1M), and cooled to 0°C, followed by addition of 2-nitrobenzyl bromide (54 mg, 4 eq.), followed by CsF (19 mg, 2 eq.). The mixture was warmed up to rt and stirred for 1h. The crude product was concentrated and purified using flash column chromatography (0 – 50% EtOAc in heptane) to afford the product (32 mg, 75%)

^{1}H NMR (400 MHz, $CDCl_3$) δ 8.10 (dd, *J* = 8.2, 1.3 Hz, 1H), 7.78 (d, *J* = 7.8 Hz, 1H), 7.71 (td, *J* = 7.5, 1.3 Hz, 1H), 7.51 (td, *J* = 7.7, 1.6 Hz, 1H), 5.60 – 5.41 (m, 2H), 5.37 – 5.30 (m, 1H), 5.21 – 5.02 (m, 2H), 4.89 – 4.61 (m, 2H), 4.24 – 3.92 (m, 5H), 3.81 (s, 3H), 2.18 (s, 3H), 2.12 (s, 3H), 2.06 (s, 3H), 1.91 (s, 3H), 1.22 (t, *J* = 7.1 Hz, 3H). **^{13}C NMR** (101 MHz, $CDCl_3$) δ 170.7, 170.4, 165.1, 155.7, 147.3, 133.9, 131.9, 129.0, 128.8, 124.9, 98.6, 98.3, 88.2, 86.3, 71.6, 71.4, 69.0, 68.8, 68.3, 63.2, 62.5, 61.4, 53.2, 47.0, 20.7, 20.7, 20.7, 20.6, 14.5. **HRMS** (ESI+) m/z calculated for $C_{28}H_{35}N_2O_{16}FNa$ 697.1868, found 697.1845. **TLC** (EtOAc/Heptane 60/40, v/v) R_f = 0.4

Experimental methods

Cell culture

HEK293 cells (CRL-1573, ATCC) and HeLa cells (CCL-2) were cultured in Dulbecco's Modified Eagle's Medium (DMEM, Gibco) supplemented with 10% fetal bovine serum (Sigma), 4 mM GlutaMAX (Gibco), and 1x penicillin-streptomycin (Gibco) at 37 °C with 5% CO_2.

UV light exposure

LEDs emitting 312 nm, 365 nm, or 412 nm light (Thorlabs) were used as light source and DMSO-dissolved **4** was radiated in plastic test tubes at 1 cm distance from the light source. HEK293 cells growing in 96-well black/clear bottom plates (Thermo Scientific) or on coverslips were exposed to 365 nm light from the bottom (ca. 1 cm distance) for the indicated time points and at maximum or partial intensity.

Lectin staining and flow cytometry

Biotinylated SiaFind Pan-specific Lectenz (Lectenz Bio) was pre-complexed with Alexa Fluor 647 conjugated streptavidin (Thermo Fisher) for 10 minutes at 4°C in 1x PBS containing 1% bovine serum albumin (PBS-BSA) at a 1:1 ratio (µg/µg), and cells were incubated with the complexes for 1 hour at 4°C. Terminal galactose residues were detected with 1 µg/ml biotinylated-peanut agglutinin (PNA) (Vector Labs) by staining the cells for 1 hour at 4°C. After washing with PBS-BSA, the cells were incubated for 20 minutes with Alexa Fluor 647 conjugated streptavidin. The cells were washed and resuspended in PBS-BSA and 10.000 cells/sample were acquired by flow cytometry using a spectral analyzer (SA3800 SONY). Mean fluorescent intensity data were used to calculate the percentage lectin binding normalized to control and all experiments were performed 2-3 times.

Immunocytochemistry

$0.7x10^5$ HEK293 cells were seeded into 6-well plates containing 24 mm coverslips coated with 0.1 mg/ml poly-L-lysine (Sigma) for 5 minutes. After 24 hours, cells were pulsed with 150 µM **4** or **5** for 4 hours and washed with medium followed by irradiation with 365 nm light for 30 seconds at maximum intensity. Post UV light-treatment, cells were cultured for 48 hours and fixed with 4% paraformaldehyde in 1x PBS for 10 min at room temperature. After washing with 1x PBS containing 0.1% v/v Tween-20 (PBS-T), cells were incubated in blocking buffer (1x PBS, 5% BSA) for 1 hour at RT. Coverslips were stained with 1 µg/ml biotinylated-PNA in 1x PBS containing 1% BSA for 1 hour at room temperature. Secondary staining was performed with 1 µg/ml streptavidin-Alexa Fluor 488 (Thermo Fisher) in 1x PBS with 1% BSA for 1 hour at room temperature in the dark. Nuclei were visualized by incubation with 1 µg/ml DAPI (Sigma Aldrich) in 1x PBS for 10 min at RT. Cells were mounted in fluorescent mounting medium (Dako), and images were acquired using a LS-900 confocal microscopy system (Leica Microsystems).

UV-energy measurement

Intensity of the UV-light treatment (mW/cm^2) was quantified using a UV power meter (Hamamatsu). The intensity was determined at 1 cm distance from the light source at max., half, and quarter capacity. Measurements were performed using no plastic (direct), through a 6-well plate bottom, a glass coverslip, and the combination. Energy was assessed over a 30 second period using average intensities.

UV-vis spectroscopy

UV-vis spectrum of **4** and **5** 150 mM stock was measured using a photospectrometer (DeNovix) with a range between 190 and 840 nm over a length of 0.5 mm.

LED-NMR

A CoolLED pE-4000 was used as a light source at 365 nm and 90% intensity. The light was transmitted into the NMR sample via a fiber optic cable terminating in a finely-sanded bare-fiber tip fitted into the inner tube of a coaxial tube. Spectra were recorded on a Bruker 500 MHz AVANCE III spectrometer equipped with a Prodigy BB cryoprobe. Spectra were recorded every 20 s.

Supplementary table and figures

Average intensity (mW/cm^2)	direct	Coverslip	6-well-plate	6-well-plate+coverslip
**Max**	225	209	181	177
**Half**	24.5	24.1	21.2	20.5
**Quarter**	4.9	4.6	4.6	4.5

Table S1 Amount of UV energy received over cell culture surface.

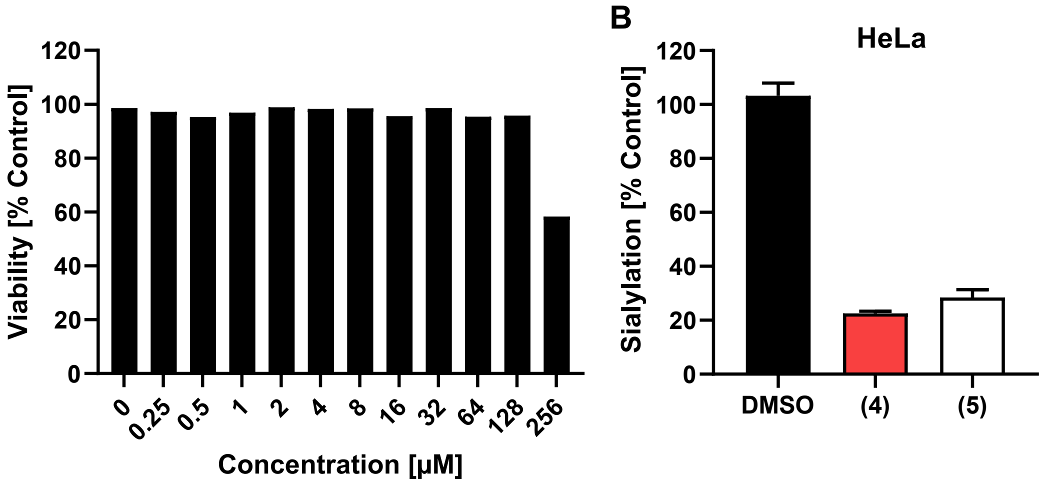

Figure S1 Effect of **4** on HEK293 cell viability and UV light mediated desialylation on HeLa cells. A) HEK293 cells were incubated for 3 days with 0-256 µM of **4** exposed to 365 nm light for 300 sec prior to addition to the cells. Viability was determined based on cell numbers and FSC/SSC appearance after 3 days of culture by flow cytometry. Bar diagram presents the percentage viability normalized to control-treated HEK293 cells of a representative experiment. B) HeLa cells were pulsed for 4 hours with 64 µM **4** pre-exposed to 365 nm light for 300 sec or unexposed **5**. After 3 days of culture, Pan-Lectenz–streptavidin Alexa Fluor 647 complexes were used to probe surface sialoglycan expression and cells were analyzed by flow cytometry. Bar diagrams show mean percentages Pan-lectenz binding (sialylation) ± SD normalized to DMSO-treated control cells of 2 representative experiments.

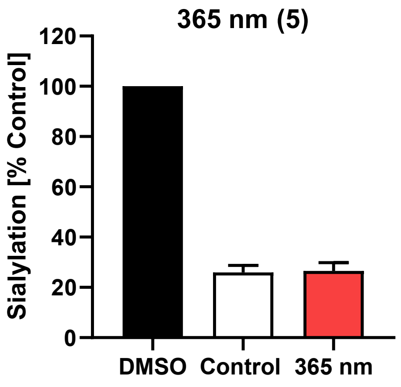

Figure S2 P-SiaFNEtoc (5) is photostable. **5** was exposed to 365 nm light for 300 sec and 64 µM was added for 3 days to HEK293 cells. Pan-Lectenz–streptavidin Alexa Fluor 647 complexes were used to probe surface sialoglycan expression and cells were analyzed by flow cytometry. Bar diagrams show mean percentages Pan-lectenz binding (sialylation) ± SD normalized to DMSO-treated cells (n=2).

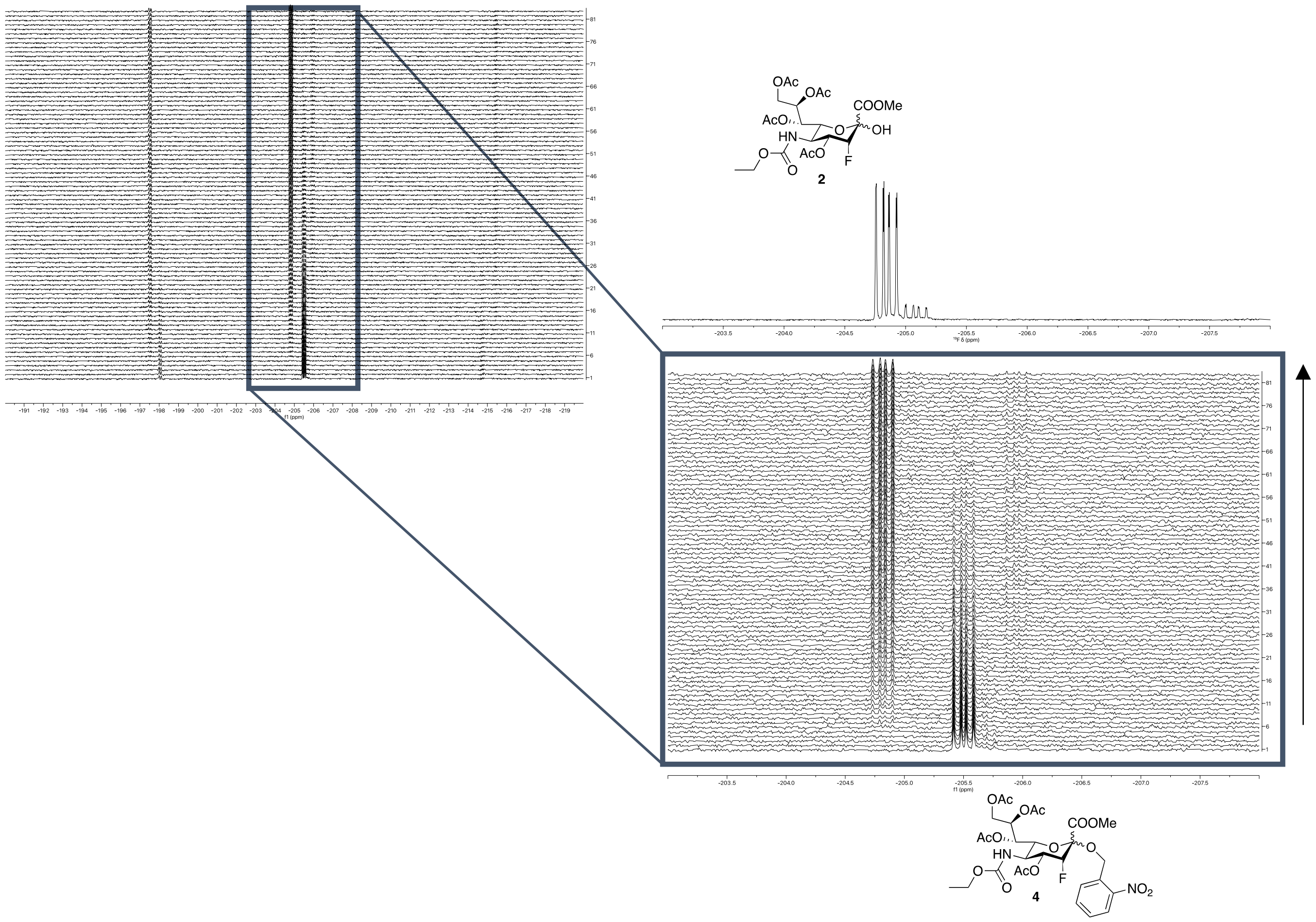

Figure S3 LED-NMR experiment of **4** to monitor cleavage of the *ortho*-nitrobenzyl ether after photoirradiation at 365nm. **4** (2mM in DMSO-D_6) was irradiated at 365nm and a ^{19}F-NMR spectrum was taken every 20 seconds. A ^{19}F-NMR spectrum of **2** in DMSO-D_6 confirms that the newly formed 19F-NMR peak corresponds to the cleaved product. Spectra were taken every 20 s for 28 minutes

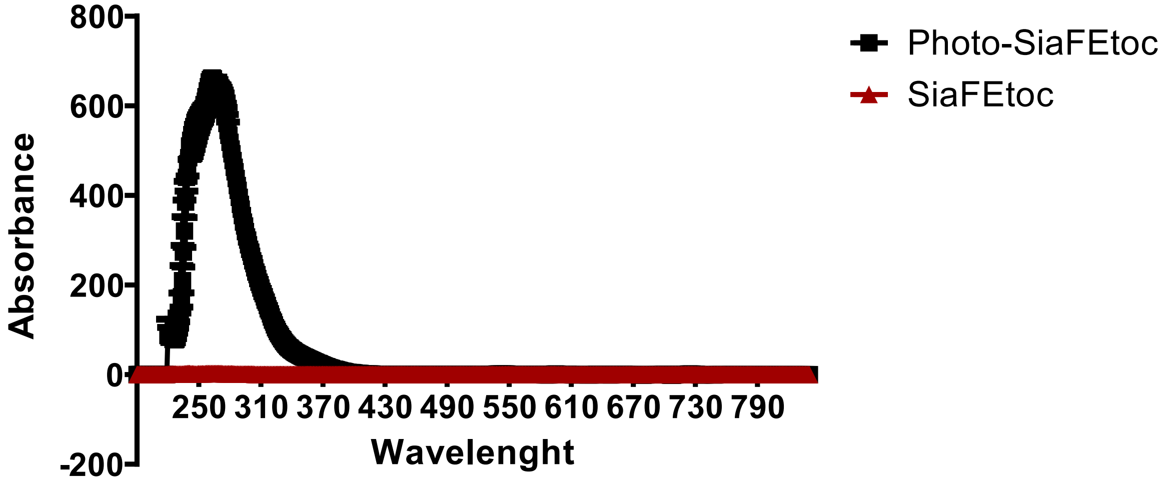

Figure S4 UV-vis spectrum of **4** (red) and **5** (black) in DMSO.

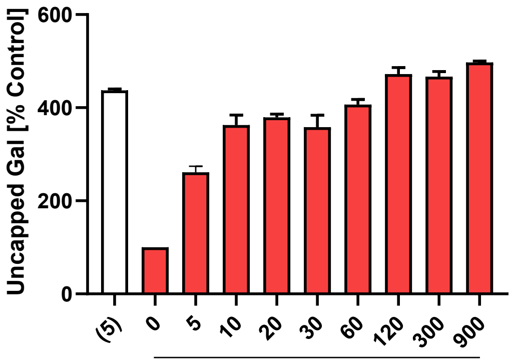

Figure S5 UV-SiaFNEtoc (**4**) was irradiated with 365 nm light for 0-900 sec prior to addition of 100 µM to HEK293 cells followed by 3 days incubation. 100 µM of 5 was added as positive control. Cells were stained with biotinylated PNA that recognizes uncapped galactose (Gal) on glycans and streptavidin-AF647 conjugate and analyzed by flow cytometry. Bar diagrams show mean percentages PNA binding (uncapped Gal) ± SD normalized to untreated control cells (n=2).

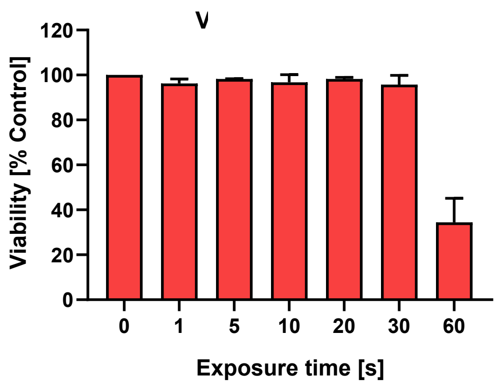

Figure S6 Effect of 365 nm irradiation on cell viability. HEK293 cells were exposed to 365 nm light (max. intensity) for different time points. After 3 days, viability was determined based on cell numbers and FSC/SSC appearance of cultures by flow cytometry. Bar diagram presents the percentage viability normalized to non-irradiated HEK293 cells of a representative experiment ± SD normalized to DMSO-treated control cells (n =3).

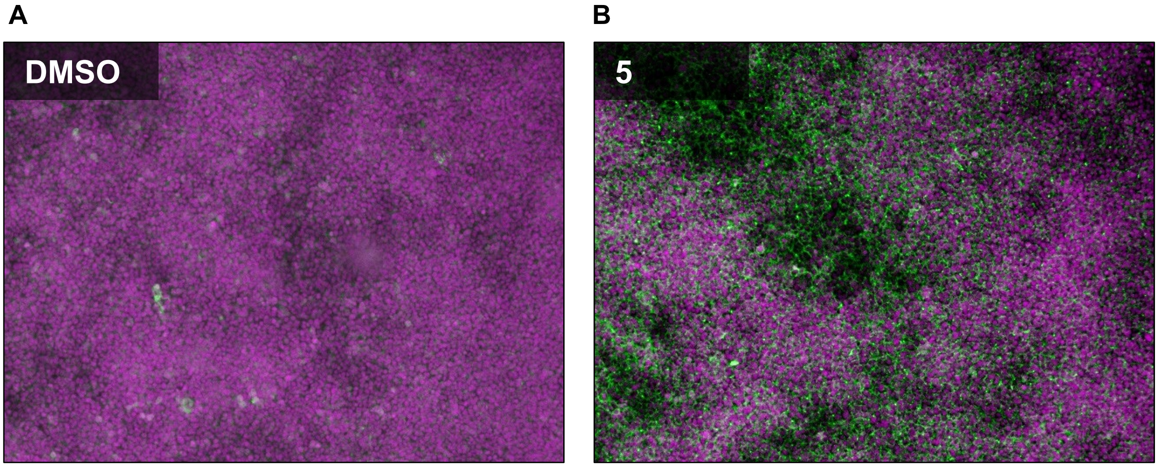

Figure S7 HEK293 cells grown on coverslips were pulsed with DMSO (A) or 150 µM **5** (B) for 4 hours and irradiated for 30 seconds with 365 nm light at max. intensity. After 24 hours, cell monolayers were stained with DAPI (magenta) and PNA (green). Representative 10x magnified microscopy images including the irradiated area are shown.

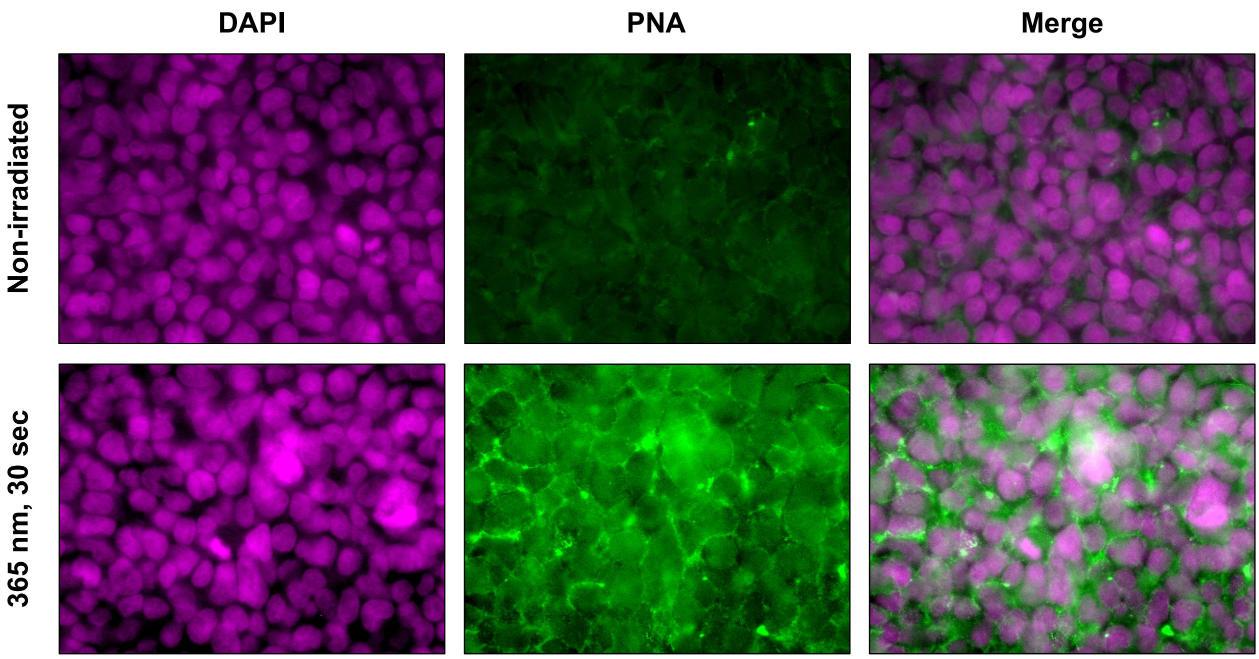

Figure S8 HEK293 cell monolayers grown on coverslips were pulsed 150 µM **4** for 4 hours and irradiated for 30 seconds with 365 nm light at max intensity. After 24 hours, cell monolayers were stained with DAPI (magenta) and PNA (green). Representative 60x magnified microscopy images show a non-irradiated region (top) and radiated region (bottom).

NMR spectra

Methyl 3,5-dideoxy-5-[(ethoxycarbonyl)amino]-3-fluoro-4,7,8,9-tetra-*O*-acetyl-D-erithro-a,β-L-manno-non-2-ulopyranosonate(2) (500MHz, $CDCl_3$)

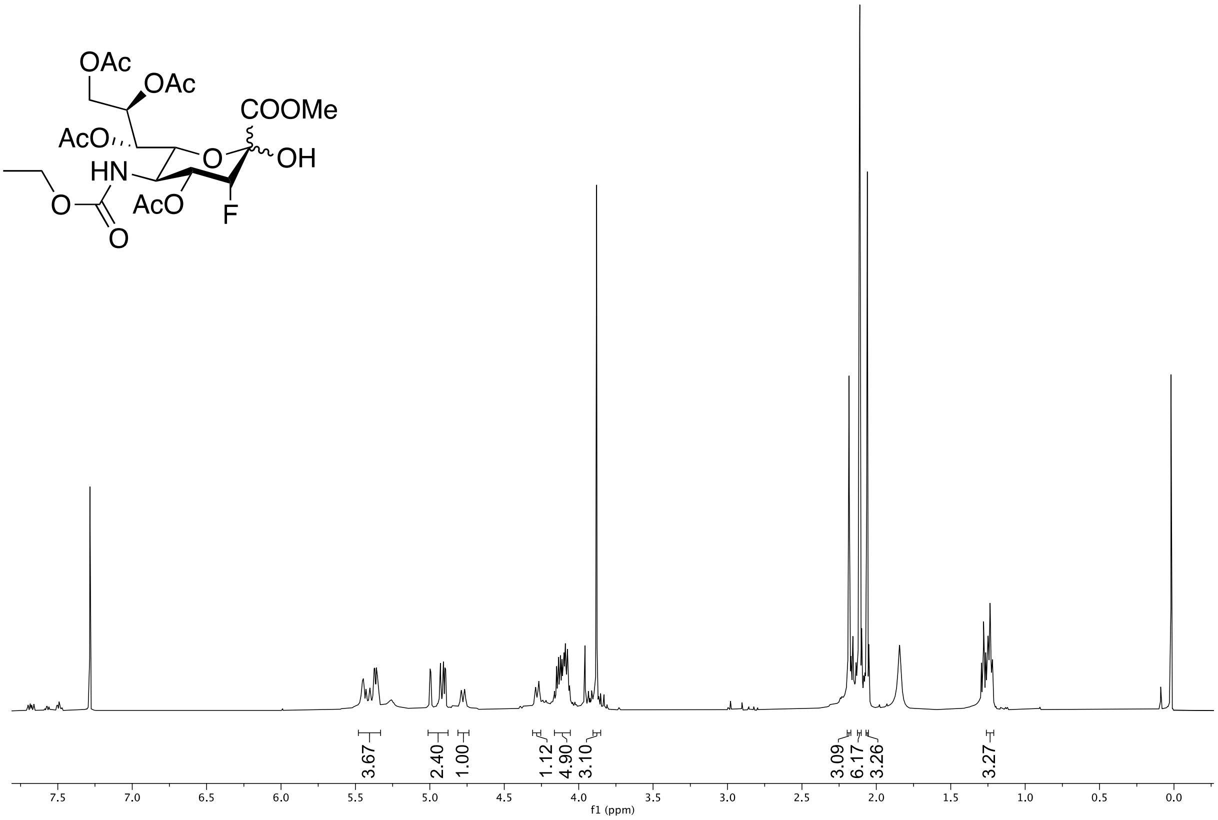

Figure S9 ^{1}H-NMR spectrum of **2**

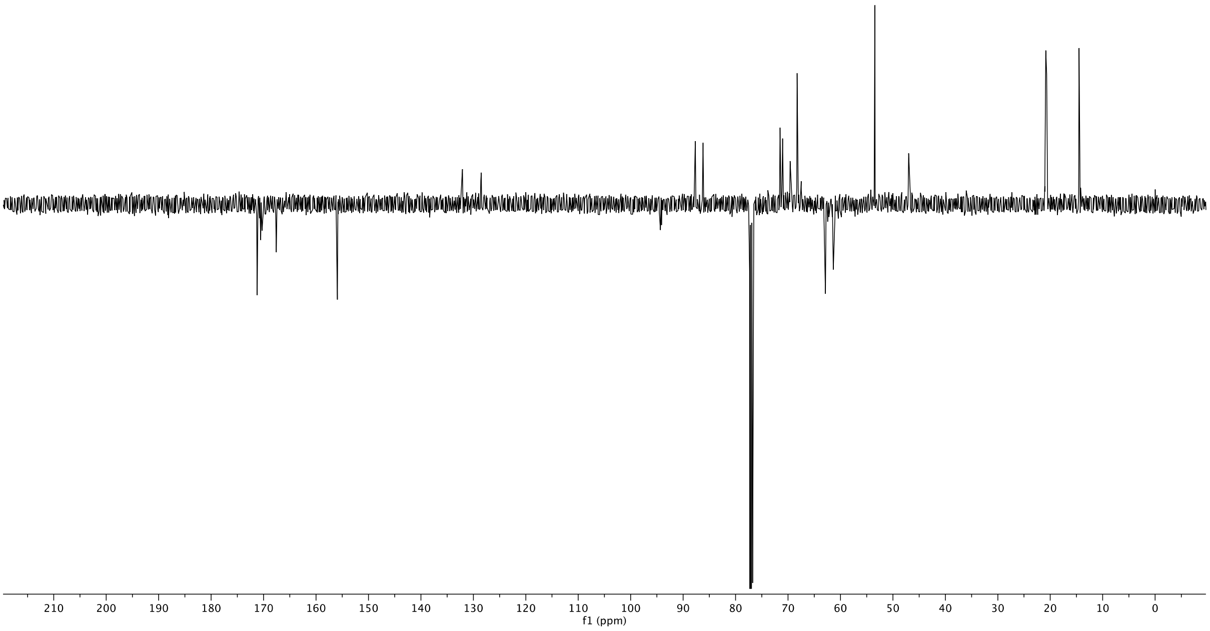

Figure S10 ^{13}C-NMR spectrum of **2**

Methyl 3,5-dideoxy-5-[(ethoxycarbonyl)amino]-3-fluoro-4,7,8,9-tetra-*O*-acetyl-2-trimethylsilyl-D-erithro-a,β-L -manno-non-2-ulopyranosonate (3) (500MHz, CDCl₃)

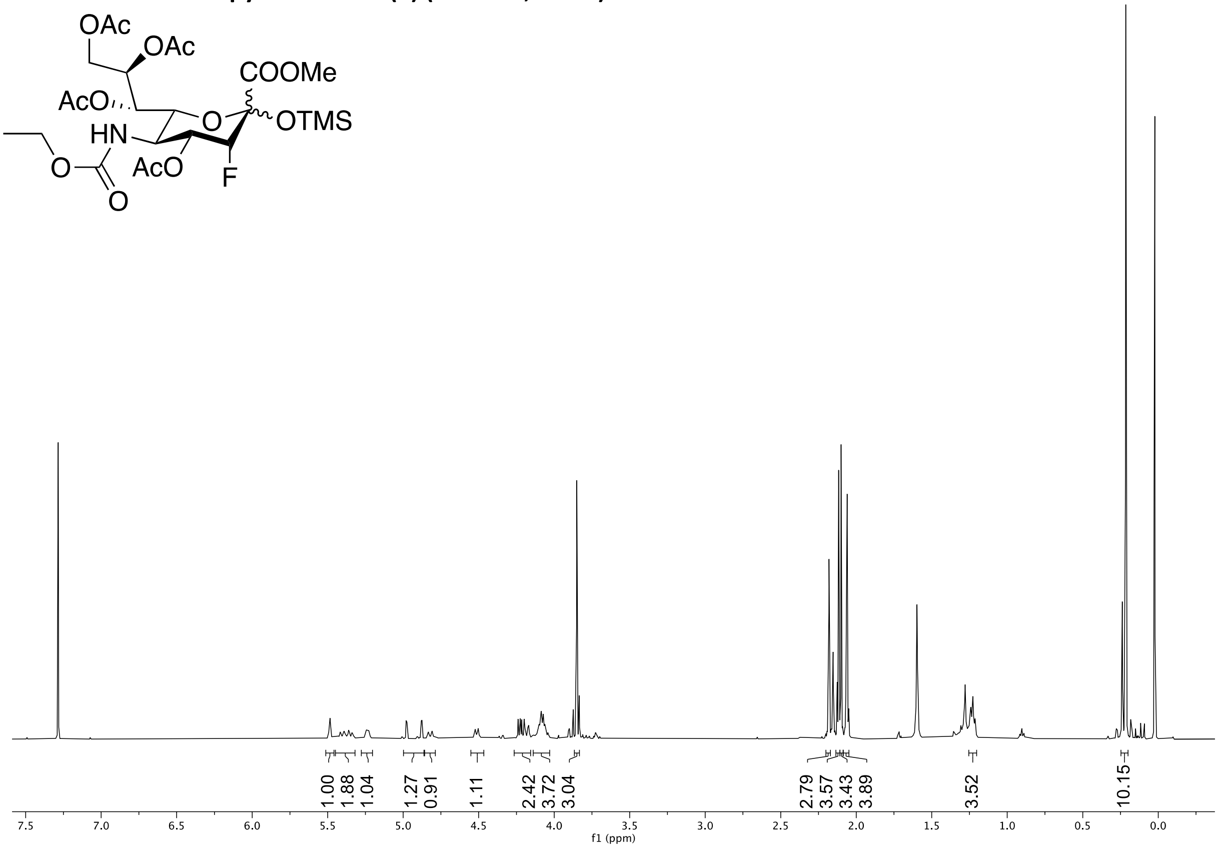

Figure S11 ^{1}H-NMR spectrum of **3**

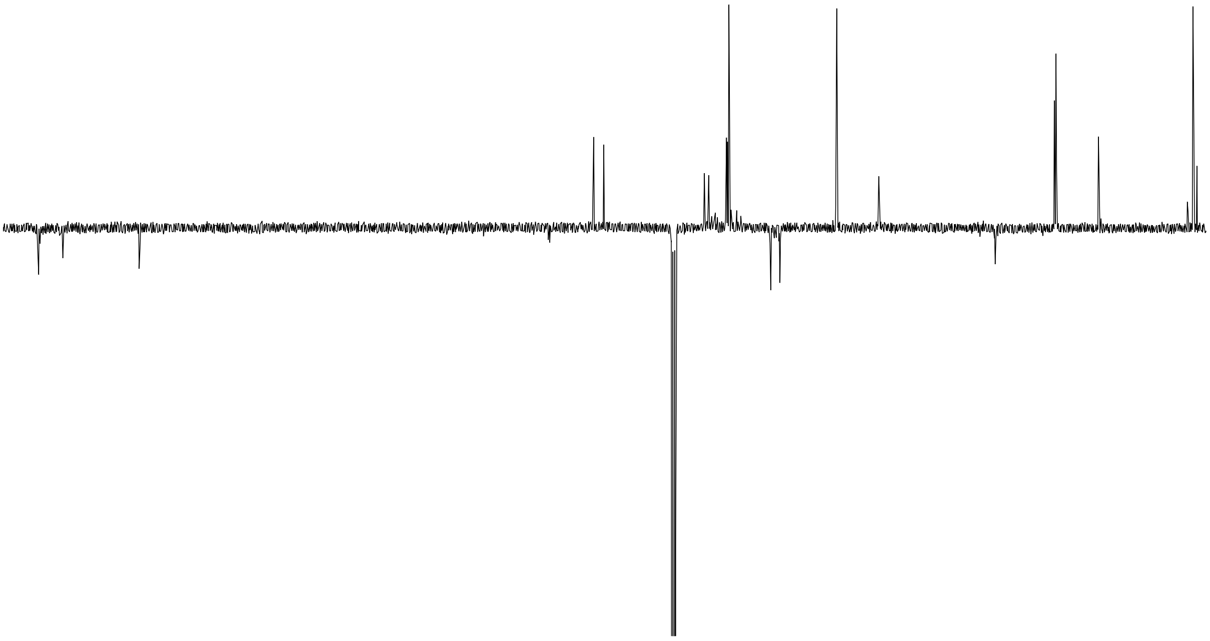

Figure S12 ^{13}C-NMR spectrum of **3**

Methyl 3,5-dideoxy-5-[(ethoxycarbonyl)amino]-3-fluoro-2-(2-nitrobenzyl)-4,7,8,9-tetra-*O*-acetyl-D-erithro-a,β-L-manno-non-2-ulopyranosonate (4) (500MHz, $CDCl_3$)

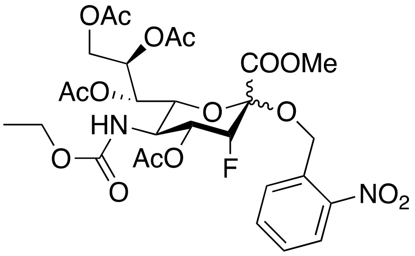

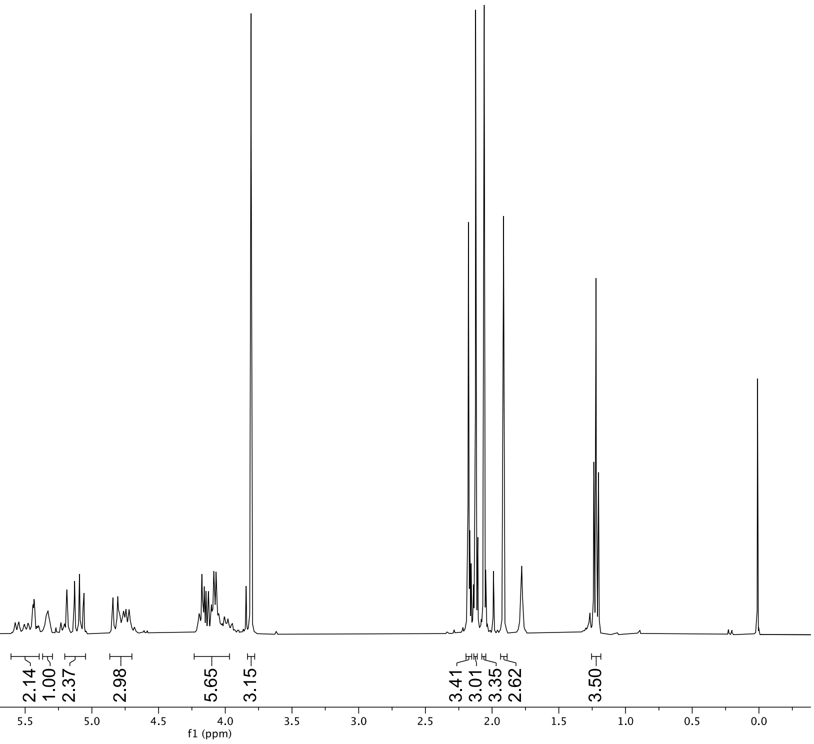

Figure S13 ^{1}H-NMR spectrum of **4**

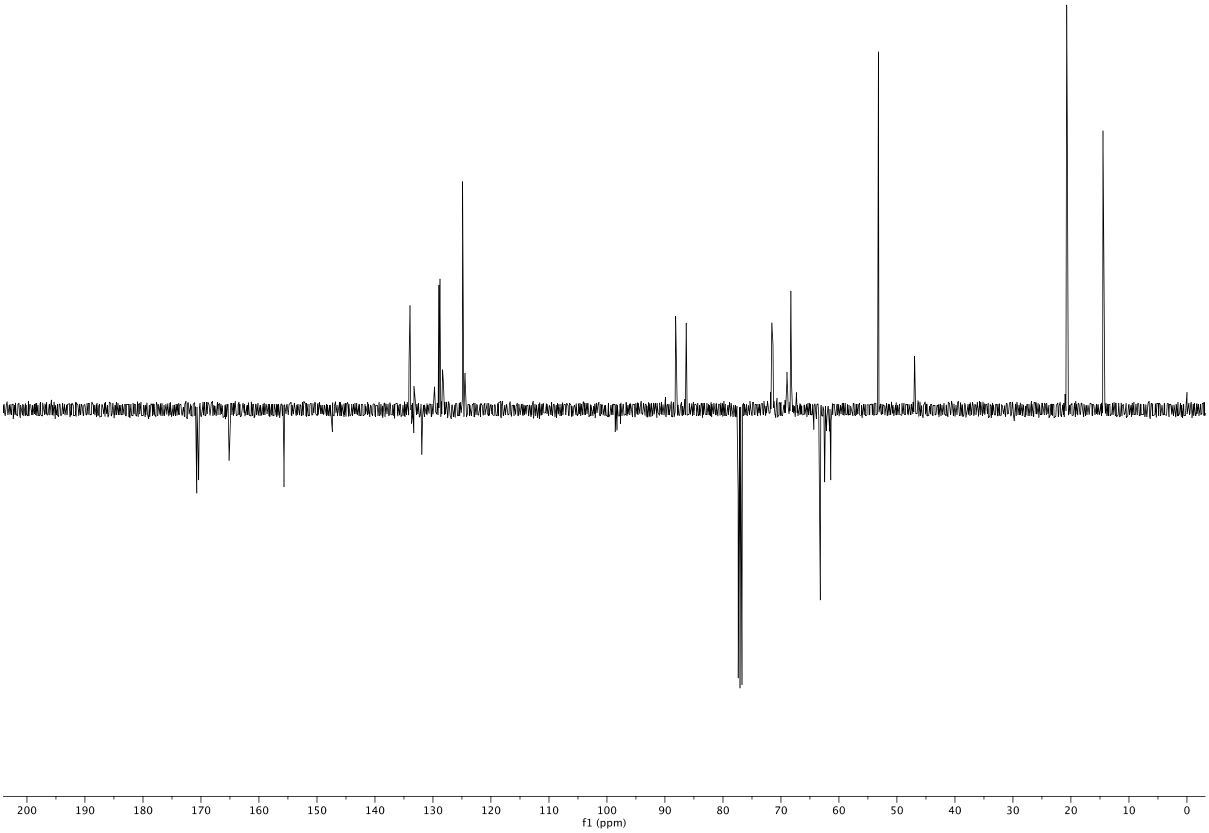

Figure S14 ^{13}C-NMR spectrum of **4**
